# Multi-Batch Consecutive Foliar Spraying Zinc–Carbon Dot Nano-Fertilizer Improving Soil Health for Bok Choy Cultivation Production

**DOI:** 10.3390/nano15221714

**Published:** 2025-11-12

**Authors:** Mengna Tao, Jiangshan Zhang, Yuying Ren, Dingge Zhang, Bingxu Cheng, Chuanxi Wang

**Affiliations:** Institute of Environmental Processes and Pollution Control, and School of Environment and Ecology, Jiangnan University, Wuxi 214122, China

**Keywords:** nano-fertilizers, zinc-carbon dot, multi-batch consecutive foliar application, soil health, sustainable agricultural production

## Abstract

This study represents the first evaluation of the effects of zinc–carbon dot nano-fertilizers (Nano-ZCDs) on the growth of Bok choy (*Brassica chinensis* L.) and soil health under multi-batch consecutive foliar spraying during three successive cultivation cycles. The results showed that relative to CK, Nano-ZCDs significantly elevated the fresh weight of Bok choy cultivation across three consecutive harvests, by 75.5 ± 1.8%, 75.1 ± 0.2%, and 74.6 ± 0.4%, respectively. Meanwhile, the nutritional value, including amino acids, vitamin C, soluble sugars, proteins, and Zn accumulation, was markedly enhanced by Nano-ZCDs. Additionally, Nano-ZCDs significantly improved soil water content, Eh, soil organic carbon, available potassium, and available phosphorus in the rhizosphere soil. It also enhanced the complexity, stability, and species richness of the bacterial community. Based on the Cornell Soil Health Assessment system, the soil health index of the Nano-ZCDs group was significantly 8.1% higher than that of the CK group. Therefore, multi-batch consecutive applying of Nano-ZCDs promoted Bok choy cultivation growth and improved its quality, without impairing soil health. This study suggests that Nano-ZCDs can be applied in agricultural production processes to promote the sustainable development of agricultural systems.

## 1. Introduction

Carbon dots (CDs), an eco-friendly nanomaterial, are increasingly used in plant ecosystems [1,2,3]. Due to their minute particle size, exceptional fluorescence properties, and commendable biocompatibility, CDs possess considerable potential in promoting crop growth and enhancing quality [4,5,6,7,8]. For instance, research by Cheng et al. demonstrated that foliar application of biomass carbon dots (B-CDs) at a dose of 10 mg/kg resulted in a significant enhancement (30–100%) of chlorophyll synthesis, as well as ferredoxin (Fd) content (40–80%) and Rubisco activity (20–110%) in lettuce leaves. Consequently, this led to an elevation of net photosynthetic rates by 130–300%, ultimately resulting in an increase in biomass of 80–150% [9]. A subsequent study posited that foliar application of 5 mg/L CDs enhanced maize growth by improving nitrogen bioavailability through the modulation of the carbohydrate metabolism and rhizosphere environment [10]. Furthermore, research has revealed that CDs can augment the absorption of nutrients by plants. In *Isatis tinctoria* L. root, CDs significantly promoted the accumulation of active components such as indigo and indigotin, increasing their levels by 128.28% and 26.63%, respectively. This enhances its medicinal quality and clinical value [11]. During post-harvest storage, applying *Salvia miltiorrhiza*-derived carbon dots (SmCDs) significantly increased the sucrose and flavonoid content of *Brassica chinensis*. After seven days, these components were elevated by 68.59% and 19.67%, respectively, compared to the control group, effectively delaying the aging process and maintaining commercial quality [12]. In recent years, there have been multiple reports focusing on the role of micronutrient-based nanofertilizers in the development of future smart agriculture and sustainable agriculture. Zn is an important essential nutrient, and the World Health Organization (WHO) recommends a daily intake of 6.7–15 mg of Zn [13]. Zn deficiency is associated with diseases such as cardiovascular damage and musculoskeletal dysfunction [14]. Biological Zn biofortification (agronomic and genetic approaches) is an effective strategy to alleviate Zn deficiency, and nanotechnology has become a key enabling technology in agriculture due to its high efficiency and targeted delivery [15]. Studies by Zhang et al. showed that foliar application of ZnO NPs could increase Zn content in rice grains, reduce the phytate–Zn ratio, and improve Zn enrichment [16]. Nevertheless, it is also necessary to note the potential environmental safety concerns of nanomaterials, such as their possible accumulation in soil ecosystems and potential impacts on non-target organisms. The pervasive adoption of CDs as novel nano-fertilizers in agriculture has rendered their entry into soil environments via direct or indirect pathways inevitable [17,18]. However, research on the potential impacts of CDs on farmland soil ecosystems remains relatively lacking, and their environmental behavior and ecological effects in this context require systematic and in-depth investigation.

Soil health is determined by its physical, chemical, and biological properties, which play a pivotal role in crop productivity [19]. Physicochemical factors, including soil texture, water content, pH level, and nutrient content, exert a direct regulatory impact on crop growth and development processes [20,21,22]. Therefore, soil health is closely linked to crop productivity and serves as a core element for sustainable agricultural development. Research has indicated that nano-fertilizers can have a substantial impact on soil health. For instance, an exposure concentration of 10 mg/L of iron nanoparticles (Fe NPs) augmented the abundance of beneficial rhizosphere microorganisms, including *Myxococcota*, *Actinobacteriota*, and *Firmicutes* phyla, enhancing bacterial community complexity [23]. Yang et al. found that foliar application of CDs induced significant changes in the rhizosphere microbiome, with the accumulation of low-molecular-weight organic compounds (e.g., succinate, pyruvate, and betaine) and the restructuring of the rhizosphere microbial community being observed. In addition, the relative abundance of certain beneficial bacterial genera was observed, including *Pseudomonas*, *Sphingomonas*, *Nitrospira*, and *Trypanosoma*. These alterations increased dissolved organic carbon (DOC) by 16.0%, available nitrogen (AN) by 33.5% and available phosphorus (AP) by 16.8%, thus leading to an improvement in soil nutrient solubility and bioavailability [24]. In most studies, the soil health index (SHI) was employed to indicate soil health and it has been successfully applied in many regions and studies for assessing soil health under different agricultural management practices [25]. In traditional SHI, principal component analysis (PCA) is a widely employed technique for defining the minimum data set (MDS) and reducing data redundancy by conducting correlation analysis between soil properties. However, the effects of nanomaterials on soil ecosystems under multi-batch consecutive foliar application are not yet fully understood, requiring urgent systematic investigation.

The existing body of research on the application potential and environmental impact assessment of CDs in agriculture is imbalanced. Furthermore, systematic investigation of their soil ecological effects under multi-batch consecutive foliar application remains largely unexplored. As a prominent vegetable crop, Bok choy is abundant in vitamins that are beneficial to human health [26]. Due to its relatively short growth period, it was selected as the model crop for this study. The study will specifically examine the following four aspects: (1) the effects of the multi-batch consecutive foliar application of Nano-ZCDs on Bok choy growth and nutritional quality; (2) the effects of the multi-batch consecutive foliar application of Nano-ZCDs on the physicochemical properties in Bok choy cultivation rhizosphere soil; (3) the effects of the multi-batch consecutive foliar application of Nano-ZCDs on microbial communities in Bok choy cultivation rhizosphere soil; (4) the evaluation of soil quality in Bok choy growing areas under the multi-batch consecutive foliar application of Nano-ZCDs.

## 2. Materials and Methods

### 2.1. Study Area and Experiment Design

This experiment was conducted in a single artificial pool (1 m × 1 m) for Bok choy cultivation in Kunshan, Suzhou (31°23′ N, 120°58′ E). The artificial pool was sited within a greenhouse (25/15 °C day/night, 18/6 h light/dark cycle). Prior to the experiment, the fundamental properties of the topsoil layer (0–20 cm) were measured as follows: Total Organic Carbon (TOC), 8.94 mg/kg; Total Carbon, 11.2 g/kg; pH, 7.05; Electrical Conductivity (EC), 0.7 ms/cm; Oxidation–Reduction Potential (Eh), 249.87 mV; Total Zn, 68.2 mg/kg.

The experiment comprised four treatment groups: Control group (CK, sprayed with an equal volume of distilled water); Zn^2+^ treatment group (Zn, sprayed with a 10 mg/kg ZnCl_2_ solution); CDs treatment group (CDs, sprayed with a 1 mg/kg CDs solution); Nano-ZCDs treatment group (Nano-ZCDs, sprayed with a 1 mg/kg Nano-ZCDs solution (Zn^2+^: CDs = 10:1)). Each treatment had three replicates. The synthesis of CDs and Nano-ZCDs followed the method reported by Ren et al. [27]. Zinc chloride anhydrous (ZnCl_2_; purity ≥ 98.0%, AR) was purchased from Titan Technologies Co., Ltd., Shanghai, China. CDs were synthesized via a hydrothermal approach. Wheat bran and ultrapure water were mixed at a mass ratio of 1:2.5, then transferred to a reactor for hydrothermal reaction at 200 °C for 10 h. Nano-ZCDs were prepared via a simple mixing method, which involved combining 105 g of ZnCl_2_, 5 g of CDs, and 390 g of ultrapure water in a beaker and leaving the mixture to stand for 2 h. The size distributions of CDs and Nano-ZCDs were 2.4 ± 0.3 and 2.3 ± 0.4 nm, which were observed by transmission electron microscopy (TEM, JEM2100F, Tokyo, Japan). CDs and Nano-ZCDs showed superior fluorescence performance, with their optimal excitation wavelength measured at 325 nm and optimal emission wavelength at 410 nm. Additionally, CDs and Nano-ZCDs exhibited a UV absorption peak at 280 and 320 nm, which was attributed to the n→π* transition. The FTIR spectra of the CDs and Nano-ZCDs revealed the presence of N-H, C-H, and C-O functional groups. The experimental design encompassed a uniform spraying protocol among all treatments. One application was conducted during the vegetative growth stage, employing a handheld sprayer to spray the leaves in a uniform manner. Bok choy leaves were sprayed with the solution (5 mL per plant) once every day for one week. The concentration of each substance applied was calculated based on the active ingredient, such as Zn^2+^. The cultivation of Bok choy was initiated in artificial ponds. Subsequent to harvesting and maturation of the initial batch, a subsequent batch was planted. Following harvesting and maturation of the second batch, the third batch was planted and subsequently yielded upon reaching maturity. All Bok choy was harvested after a cultivation period of 20 days in artificial ponds.

### 2.2. Bok Choy Cultivation Sample Testing

The methods of determining the physiological characteristics and quality indicators in Bok choy cultivation are as follows: (1) after harvesting, the samples were washed and dried with filter paper; (2) an electronic balance (OHAUS AX224ZH/E) was used to weigh fresh weight. Its nominal accuracy is 0.01 g. To measure dry weight, fresh samples were placed in a 105 °C oven (Jinghong, Shanghai) for 30 min and then dried at 60 °C until they reached a constant weight. The temperature calibration of the oven was completed using a certified standard mercury thermometer (accuracy: ±0.1 °C). The amino acid, vitamin C, soluble sugar, and protein content of Bok choy were examined using relevant assay kits provided by the Beijing Solarbio Company, with the corresponding catalog numbers being BC1575, BC1230, BC0030, and BC3185. All detection procedures were performed in accordance with the instructions provided with the kits. Absorbance was measured using a microplate reader (Thermo Fisher Scientific, Waltham, MA, USA), and the content of each index was then calculated according to the formula supplied with the kits.

The dried samples were grinded into a uniform powder for subsequent use. A measurement of 25.0 mg of sample was weighed accurately and then put into a digestion vessel. Sequentially, 3 mL of ultrapure water and nitric acid (HNO_3_, AR) were added. Then, microwave digestion system (CEM, MARS 6, Matthews, NC, USA) was used to perform a digestion program. Then, the solution was filter through a 0.22 µm microporous membrane and diluted with ultrapure water to 50 mL. Finally, the inductively coupled plasma mass spectrometer (ICP-MS, iCAP-TQ, Thermo Fisher, Bremen, Germany) was employed to determine Zn content. The detection limit (LOD) was 0.004 μg/L and R^2^ for detection of Zn by ICP-MS was 0.9998. Each sample had five replications.

### 2.3. Soil Sample Collection and Determination of Physicochemical Parameters

The soil was collected by carefully uprooting Bok choy and collecting the soil adhering to the roots [28]. The soil between roots was transferred to 10 mL sterile tubes and placed in a liquid nitrogen tank. It was then quickly returned to the laboratory and stored at −80 °C for soil microbial community analysis. The remaining fresh soil samples were air-dried at room temperature for soil physicochemical properties investigation.

The available phosphorus (AP), available nitrogen (AN), and soil organic carbon (SOC) of the soil were analyzed by the published methods [24]. The contents of available potassium (AK) were analyzed using assay kits (Beijing Solare Technology Co., Ltd., Beijing, China). The pH, EC, and Eh were determined as follows: 2 g soil samples and 10 mL ultrapure water were placed in 50 mL centrifuge tubes and shaken in a shaker (200 r/min) for 1 d. Soil pH was tested with a pH meter (OHAUSST310, Parsippany, NJ, USA), and soil EC and Eh were measured with SevenExcellence (MettlerToledo, Greifensee, Canton of Zurich, Switzerland). TOC was examined using Different TOC Selection (Elemental, Langenselbold, Hesse, Germany).

### 2.4. Soil Microbial Community Analysis

To investigate the abundance and composition of soil microorganisms, the prepared amplicon libraries were analyzed by PE250 using the Illumina Nova 6000 platform (Guangdong Meiji Gene Biotechnology Co., Ltd., Guangzhou, China). The total DNA from soil microorganisms was first extracted, and its purity and concentration were detected using Thermo NanoDrop One. Genomic DNA was then subjected to PCR amplification using specific primers with barcodes and TaKaRa Premix Taq Version 2.0 (TaKaRa Biotechnology Co., Ltd., Dalian, China) to identify bacterial diversity. After quality filtering (removing reads with Q-score < 20 and adapter contamination), each soil sample yielded an average of 28,500–32,200 high-quality. The QIIME2 (version 2022.11) pipeline was used for quality control, diversity analysis, and sequence classification. The ITS1 region primers (ITS5-1737F and ITS2-2043R) were identified for fungal diversity. Bacterial 16S rRNA gene sequences (amplified from the V4 to V5 region) were aligned against the SILVA database (version 138). Fungal ITS1 region sequences were annotated using the UNITE database (version 10.0).

### 2.5. Calculation of Soil Health Index

Soil health is commonly overlooked in the evaluation of food crop production systems. In this study, we applied the Cornell Soil Health Assessment (CSHA) scoring method to systematically evaluate soil function using multidimensional indicators [29]. Key parameters such as water content, soil organic carbon (SOC), and Zn content were selected. Data were acquired using a standardized laboratory analysis and indicators were normalized to generate a composite health score [30]. Principal component analysis (PCA) was used to investigate the effect of crop diversification on soil health [31]. The analysis was completed using SPSS 26 software. Firstly, the data applicability was verified by the Kaiser–Meyer–Olkin (KMO) test and Bartlett’s test of sphericity. The principal components were extracted with eigenvalues >1 (Kaiser’s criterion) and combined with Varimax rotation to maximize the variance and optimize the factor loading matrix. The cumulative variance contribution was filtered to >80%. The key soil parameters significantly associated with crop diversity were identified by rotated loadings (absolute value > 0.5). The results are as follows. The weighting factor for Eh is 0.157, for AK is 0.193, for AP is 0.18, for AN is 0.08, and for W is 0.162. The weighting factor for SOC is 0.228. The overall soil health score was calculated as a weighted average of all individual CSHA scores, using the following formula [30,31]:

(1) $$\mathrm{Soil}\;\mathrm{health}\;\mathrm{score}=\frac{\left(A_{1}\times w_{1}\right)+\left(A_{2}\times w_{2}\right)+\ldots +(A_{n}\times w_{n})}{w_{1}+w_{2}+\ldots +w_{n}}$$

where *A* is the CSHA score for each individual soil indicator, and *w* is the weighting factor for each soil indicator.

### 2.6. Data Analysis

Differences between treatments were analyzed using one-way ANOVA (*p* < 0.05, Fisher LSD test) with Origin Pro 2021b software. Principal component analysis (PCA) was performed using SPSS Statistics 26.0 software. All data were expressed as the mean ± standard deviation (SD). All treatments had at least three replications.

## 3. Result and Discussion

### 3.1. Effects of Multi-Batch Consecutive Foliar Application Nano-ZCDs on Bok Choy Growth

**Figure 1:**
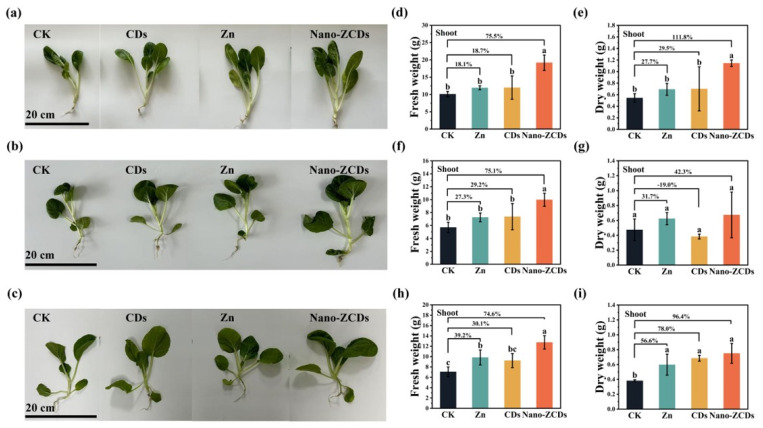
The growth of Bok choy in three batches after exposure to Zn, CDs, and Nano-ZCDs. Effect photos (**a**–**c**); shoot fresh weight (**d**) and dry weight (**e**) in the first batch of Bok choy cultivation; shoot fresh weight (**f**) and dry weight (**g**) in the second batch of Bok choy cultivation; shoot fresh weight (**h**) and dry weight (**i**) in the third batch of Bok choy cultivation. Results were shown as means ± SD. Different letters indicate significant differences among treatments (*p* < 0.05, LSD, *n* = 3).

Compared to the control (CK), foliar application Nano-ZCDs significantly promoted Bok choy growth across all experimental batches, with superior performance relative to individual Zn or CDs treatments (Figure 1). Specifically, the Zn, CDs, and Nano-ZCDs treatments in Batch 1 increased shoot fresh weight by 18.1%, 18.7%, and 75.5% compared to CK (Figure 1d), while shoot dry weight increased by 27.7%, 29.5%, and 111.8% (Figure 1e). Notably, the Nano-ZCDs treatment significantly increased both fresh and dry biomass. In Batch 2, the Zn, CDs, and Nano-ZCDs treatments elevated shoot fresh weight by 27.3%, 29.2%, and 75.1%, respectively (Figure 1f). However, the shoot dry weight responses varied. Zn and Nano-ZCDs treatments had positive effects, resulting in increases of 31.7% and 42.3%, whereas CDs treatment resulted in a 19.0% reduction (Figure 1g). Only the Nano-ZCDs treatment exhibited a statistically significant growth-promoting effect in this batch (*p* < 0.05). In Batch 3, shoot fresh weight increased by 39.2%, 30.1%, and 74.6%, and dry weight rose by 56.6%, 78.0%, and 96.4% (Figure 1h,i), respectively. Nano-ZCDs showed the most pronounced enhancement again. Overall, these findings indicate that Nano-ZCDs outperform single Zn or CDs formulations in promoting Bok choy growth, with its efficacy consistently superior across all tested batches. These results align with those reported by Ren et al., who revealed that Nano-ZCDs exhibited the most significant growth-stimulating effect on lettuce (*Lactuca sativa* L.) compared to Zn and CDs fertilizers [27].

### 3.2. Effects of Multi-Batch Consecutive Foliar Application Nano-ZCDs on the Quality and Zn Content in Bok Choy

**Figure 2:**
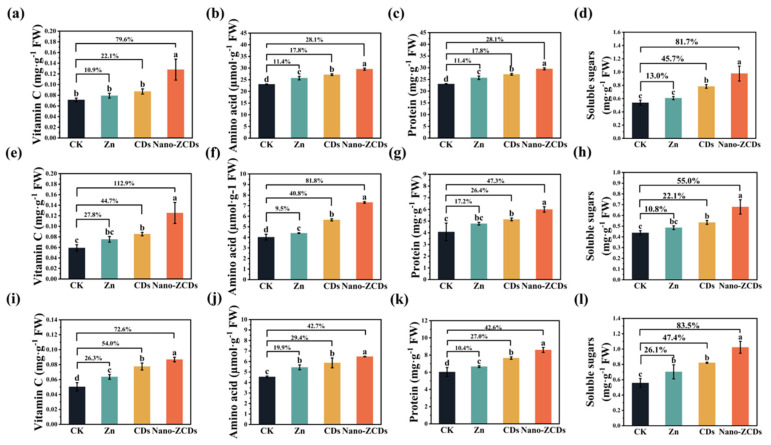
The quality of Bok choy in three batches in response to Zn, CDs, and Nano-ZCDs. Changes in amino acid (**a**), vitamin C (**b**), protein (**c**), and soluble sugar (**d**) contents in the first batch grown; changes in amino acid (**e**), vitamin C (**f**), protein (**g**), and soluble sugar (**h**) contents in the second batch grown; changes in amino acid (**i**), vitamin C (**j**), protein (**k**), and soluble sugar (**l**) contents in the third batch grown. Results were shown as means ± SD. Different letters indicate significant differences among treatments (*p* < 0.05, LSD, *n* = 3).

**Figure 3:**
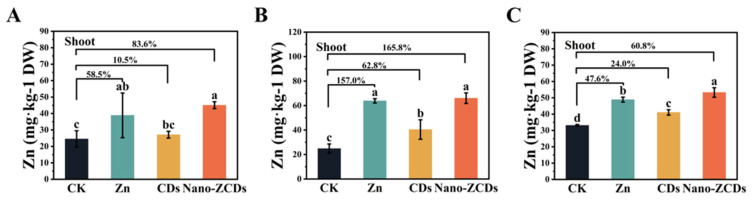
Effect of the Zn, CDs, and Nano-ZCDs on the Zn content in Bok choy shoots. (**A**) Zn content of Bok choy shoots in the first batch grown; (**B**) Zn content of Bok choy shoots in the second batch grown; (**C**) Zn content of Bok choy shoots in the third batch grown. Results were shown as means ± SD. Different letters indicate significant differences among treatments (*p* < 0.05, LSD, *n* = 3).

Compared to CK, foliar applications of Zn, CDs, and Nano-ZCDs significantly increased the levels of amino acids, vitamin C (Vc), protein, and soluble sugars in Bok choy across all three experimental batches, with Nano-ZCDs consistently demonstrating the most pronounced effects. In Batch 1, Nano-ZCDs treatment substantially enhanced Vc, amino acid, protein, and soluble sugar amounts by 79.6%, 62.4%, 28.1%, and 81.7%, respectively (Figure 2a–d). Corresponding increments in Batch 2 were 81.8%, 112.9%, 47.3%, and 55.0%, while Batch 3 showed increases of 42.7%, 72.6%, 42.6%, and 83.5% (Figure 2e–l). All these nutrient levels were significantly higher in Nano-ZCDs treatment groups than those in CK and other treatment groups. Amino acids, as essential plant nutrients, contribute to flavor and aroma formation and participate in plant defense mechanisms [32,33]. For example, valine, leucine, proline, and tyrosine function as alternative respiratory substrates under different stresses [32]. Vc is a strong antioxidant that stops damage caused by reactive oxygen species [34,35]. Vegetables provide a critical dietary source of Vc, and a lack of it can lead to various diseases, making vegetable consumption an effective and accessible means of supplementation [36]. Proteins and soluble sugars are pivotal in maintaining osmotic balance [37,38]. Additionally, Nano-ZCDs treatment significantly elevated Zn accumulation in Bok choy shoots, with Zn content increasing by 83.6%, 165.8%, and 36.6% in Batches 1–3, respectively (Figure 3A–C). The increased protein content might be attributed to the enhanced delivery method of zinc, since zinc foliar application is proven to significantly enhance protein content in plants [39]. This exceeded the accumulation levels observed with Zn or CDs treatments alone. Taken together, these findings evidenced that Nano-ZCDs effectively enhance the primary nutritional quality components of Bok choy and exhibit a remarkable Zn-enrichment effect, outperforming single Zn or CDs formulations in overall efficacy.

### 3.3. Effects of Multi-Batch Consecutive Foliar Application of Nano-ZCDs on Soil Physico-Chemical Properties

**Figure 4:**
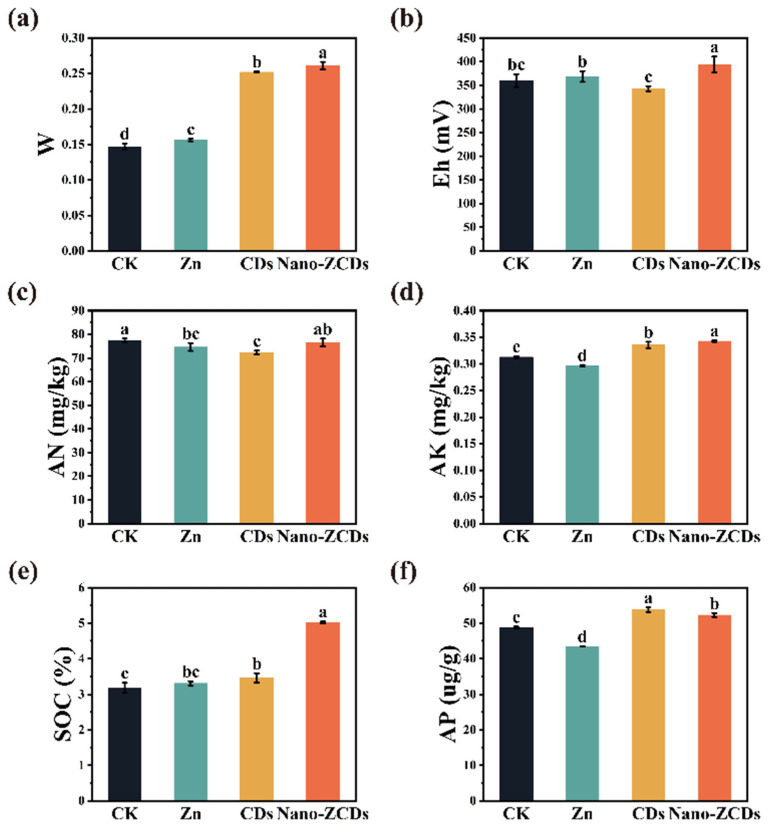
Effect of the Zn, CDs, and Nano-ZCDs on the soil properties after the third Bok choy harvest. (**a**) W; (**b**) Eh; (**c**) AN; (**d**) AK; (**e**) SOC; (**f**) AP. Results were shown as means ± SD. Different letters indicate significant differences among treatments (*p* < 0.05, LSD, *n* = 3).

The physicochemical characteristics of soil are pivotal elements affecting soil health and quality, directly impacting plant productivity and root growth [21]. As illustrated in Figure 4a,b, the Nano-ZCDs treatment significantly increased soil water content (W) and Eh by 77.5% and 9.4%, respectively, compared with the CK. Eh serves as an important indicator of soil redox processes, with higher values indicating a stronger oxidizing environment and lower values indicating a stronger reducing environment [40]. Studies have shown that under strongly oxidizing conditions, certain substances such as organic matter, carbonates, phosphates, and free silica can inhibit the formation of iron nuclei. This promotes the rapid oxidation of Fe (II) [41,42]. The formation of active iron is facilitated, with effective nutrients being provided for plants. In addition, treatment with Nano-ZCDs significantly increased the contents of AK and AP by 9.5% and 7.0%, respectively (Figure 4d,f). However, the content of AN showed a slight decline of 1.2% in Nano-ZCDs treatment (Figure 4c). This phenomenon may be attributed to the capacity of nanomaterials to enhance crop nitrogen uptake. Liu et al. discovered that nano-iron oxide (Fe_3_O_4_) decreased total nitrogen content in the rhizosphere. This phenomenon could be attributed to the stimulatory effect of nano-iron oxide (Fe_3_O_4_) on nitrogen uptake in ryegrass, which is associated with an increase in soil pH [43]. This reduction was evidence of efficient nitrogen utilization, meaning it will not have a long-term impact on Bok choy productivity. SOC is a key indicator of soil health, contributing to structural stability, nutrient availability, water retention capacity, and microbial activity and abundance [44,45]. The SOC content in the Nano-ZCDs-treated group was significantly higher than that in the CK group, with an increase of 57.7% (Figure 4e). In summary, Nano-ZCDs treatment can markedly improve the soil physicochemical properties, enhancing its redox capacity and nutrient retention function. Nevertheless, although most indicators exhibit positive responses, variations in some nutrient indicators still require attention to maintain soil health.

### 3.4. Effects of Multi-Batch Consecutive Foliar Application of Nano-ZCDs on Soil Microbial Community

**Figure 5:**
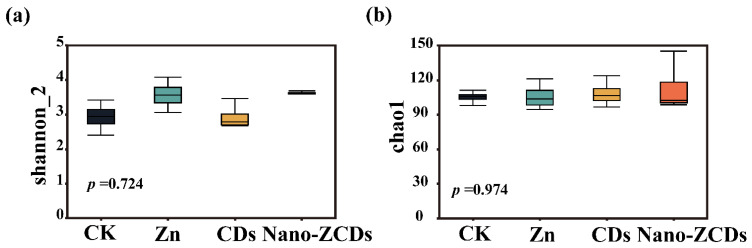

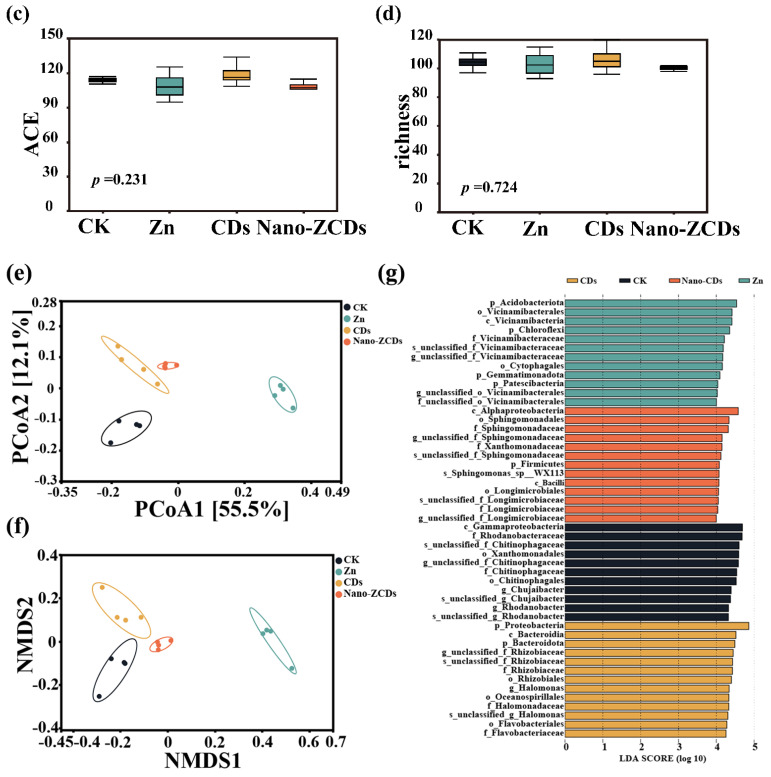
Effect of the Zn, CDs, and Nano-ZCDs on the soil microbial community diversity after the third Bok choy harvest. (**a**) Shannon index; (**b**) Chao 1 Index; (**c**) ACE index; (**d**) microbial abundance. (**e**) Principal coordinates analysis (PCoA) and NMDS analysis (**f**) of the soil microbial communities; (**g**) results of LEfSe analysis of the soil microbial community. Error bars represent standard deviation of four replicates.

Soil microbes are crucial for nutrient cycling, pathogen resistance, maintaining soil ecology, and promoting crop growth [46]. The Shannon and Chao1 indices are crucial for characterizing the α-diversity of the soil microbial community and assessing soil health status [47]. These indices reflect the complexity, stability, and species richness of microbial communities [48]. As shown in Figure 5a,b, Nano-ZCDs treatment exerted positive effects on the α-diversity indices of the microbial community. Among them, the Shannon index represents species diversity and abundance, while the Chao1 index focuses more on indicating species richness [49]. The rhizosphere microbial community comprises a diverse range of potential plant pathogens and antagonistic microorganisms, thereby exerting a significant influence on plant health and soil disease suppression [50]. Compared with the CK, Nano-ZCDs treatment enhanced the microbial community richness (Figure 5c,d). The PCoA analysis further indicated that the soil microbial β-diversity was significantly affected under various treatments (Figure 5e). Also, the results of the NMDS analysis based on Bray–Curtis distances indicated that the soil microbial community structure was indeed affected among different treatments (Figure 5f). These results showed that Nano-ZCDs could affect the β-diversity of soil microorganisms. This change may be associated with the increases in SOC content and W content under Nano-ZCDs treatment. Alterations in these physicochemical properties have been demonstrated to drive the evolution of rhizosphere microbial community structure and diversity [51,52]. There were 1813 taxa found to be unique in microbial communities (*p* < 0.05), respectively. Accordingly, 49 taxa were identified as the top bulk soil taxa with LDA of > 4.0 (Figure 5g). The phylum *Firmicutes*, its order *Sphingomonadales* and *Longimicrobiales*, and the genera of *Sphingomonadaceae* and *Longimicrobiaceae* were the main biomarkers in the Nano-ZCDs group, indicating that *Firmicutes* had the most significant effect following Nano-ZCDs treatment (Figure 5g). *Firmicutes* taxa in the rhizosphere play a critical role in facilitating organic C decomposition and plant nutrient uptake [53]. Khan et al. discovered inoculation of a pea plant with *Firmicutes* significantly increased the amount of chlorophyll, protein, starch, proline, and carbohydrates [54]. In summary, multi-batch consecutive foliar application of Nano-ZCDs contributed to maintaining the diversity and abundance of soil microbial communities, consequently exerting a positive impact on the ecological functions of soil microorganisms.

### 3.5. Soil Health Assessment Under Multi-Batch Consecutive Foliar Application of Nano-ZCDs

**Figure 6:**
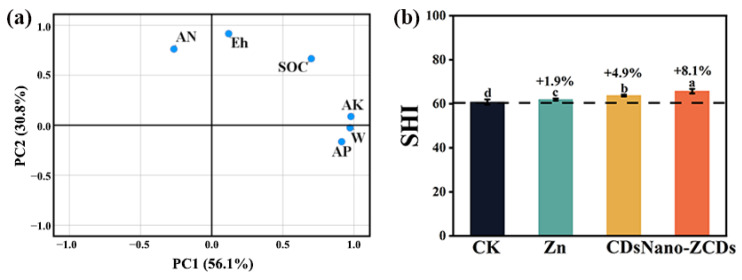
Soil health assessment in Zn, CDs, and Nano-ZCDs treatment. (**a**) PCA of six key soil trait values, including SOC, AN, AP, AK, Eh, and W; (**b**) SHI under different treatment. Results were shown as means ± SD. Different letters indicate significant differences among treatments (*p* < 0.05, LSD, *n* = 3).

Six soil properties, including SOC, AN, AP, AK, Eh, and W, were analyzed using post-harvest rhizosphere soil samples to evaluate the accumulated impacts of fertilization variation on soil health (Figure 6). Principal component analysis (PCA) was employed to select representative indicators of soil health (Figure 6a). The first two principal components accounted for 86.9% of cumulative percentage variability in rhizosphere soil health (PC1 = 56.1%, PC2 = 30.8%), and the eigenvalues of PC1 and PC2 were 3.37 and 1.85. Soil health scores (Cornell Soil Health Assessment Scoring) were influenced by different fertilization types (Figure 6b). Compared with the CK group, the rhizosphere soil health scores increased by 1.9% ± 0.6, 4.9% ± 0.6, and 8.1% ± 0.2 in the Zn, CDs, and Nano-ZCDs treatment groups, respectively. In conclusion, this evidence implied that Nano-ZCDs could support greater soil health compared to Zn and CDs.

### 3.6. Limitations, Challenges, and Prospects

Despite the present study providing valuable insights into the effects of the multi-batch consecutive foliar application of Nano-ZCDs on Bok choy growth and rhizosphere soil health, several limitations should be acknowledged to guide future research. The test crop in this study has a short growth cycle, which may fail to capture multi-year long-term cumulative effects, such as the persistent residues of Nano-ZCDs in soil and seasonal variations in microbial communities. Additionally, the environmental safety assessment of Nano-ZCDs remains incomplete. While the present study confirmed that soil health remained stable following the application of Nano-ZCDs and no excessive Zn accumulation occurred in Bok choy, it did not explore the migration and transformation of Nano-ZCDs within the soil–plant system or their potential bioaccumulation in the food chain. This study exclusively focused on Bok choy, and the universality of Nano-ZCDs for other crop types (e.g., cereal crops such as wheat and rice) remains to be verified.

Future research should (1) establish 2–3 years positioning trials; (2) track Nano-ZCDs’ fate and test non-target ecotoxicity; (3) expand the scope of test crops.

## 4. Conclusions

In short, the multi-batch consecutive application of Nano-ZCDs could not only maintain the stable accumulation of Bok choy biomass but also enhance its quality. Furthermore, it also significantly improved the physicochemical properties and microbial community diversity of rhizosphere soil. Based on the evaluation results of the Cornell Soil Health Assessment framework, multi-batch consecutive application of Nano-ZCDs leads to no decline in soil quality, with the soil health index remaining at a favorable level. The Nano-ZCDs used in this study were synthesized via a green hydrothermal approach without toxic additives, providing a fundamental safety basis for agricultural application. The multi-batch consecutive foliar application tested here provided three lines of evidence in support of Nano-ZCDs’ environmental safety. Firstly, no excessive Zn accumulation was detected in Bok choy shoots, meeting leafy vegetable food safety thresholds. Secondly, soil physicochemical properties remained stable with no adverse changes, confirming no disruption to the soil’s inherent abiotic functions. Thirdly, the relative abundance of beneficial microbial taxa (e.g., phylum *Firmicutes*) linked to plant growth and nutrient cycling was significantly increased. Collectively, these findings verify the environmental safety of Nano-ZCDs under the evaluated multi-batch consecutive foliar application conditions. To balance their agronomic benefits and environmental safety, future research should evaluate the migration and potential bioaccumulation of Nano-ZCDs within soil–plant systems. Therefore, the application of Nano-ZCDs has the potential to be an effective and environmentally friendly agronomic practice that helps improve the quality of continuously cropped soil and enhance crop yield and quality. To comprehensively validate its long-term efficacy, environmental safety, and general applicability across diverse soil types and climatic conditions, further multi-year field trials are recommended.

## Data Availability

The original contributions presented in this study are included in the article. Further inquiries can be directed to the corresponding authors.
